# Insertion Defects of Mitochondrially Encoded Proteins Burden the Mitochondrial Quality Control System

**DOI:** 10.3390/cells7100172

**Published:** 2018-10-17

**Authors:** Braulio Vargas Möller-Hergt, Andreas Carlström, Tamara Suhm, Martin Ott

**Affiliations:** Department of Biochemistry and Biophysics, Stockholm University, SE-10691 Stockholm, Sweden; braulio.vargas.moller-hergt@dbb.su.se (B.V.M.-H.); andreas.carlstrom@dbb.su.se (A.C.); tamara.suhm@dbb.su.se (T.S.)

**Keywords:** mitochondria, mitochondrial quality control, membrane protein insertion, membrane-bound AAA proteases

## Abstract

The mitochondrial proteome contains proteins from two different genetic systems. Proteins are either synthesized in the cytosol and imported into the different compartments of the organelle or directly produced in the mitochondrial matrix. To ensure proteostasis, proteins are monitored by the mitochondrial quality control system, which will degrade non-native polypeptides. Defective mitochondrial membrane proteins are degraded by membrane-bound AAA-proteases. These proteases are regulated by factors promoting protein turnover or preventing their degradation. Here we determined genetic interactions between the mitoribosome receptors Mrx15 and Mba1 with the quality control system. We show that simultaneous absence of Mrx15 and the regulators of the i-AAA protease Mgr1 and Mgr3 provokes respiratory deficiency. Surprisingly, mutants lacking Mrx15 were more tolerant against proteotoxic stress. Furthermore, yeast cells became hypersensitive against proteotoxic stress upon deletion of *MBA1*. Contrary to Mrx15, Mba1 cooperates with the regulators of the m-AAA and i-AAA proteases. Taken together, these results suggest that membrane protein insertion and mitochondrial AAA-proteases are functionally coupled, possibly reflecting an early quality control step during mitochondrial protein synthesis.

## 1. Introduction

Mitochondria contain their own genetic system [1]. The organelle houses factors to transcribe and translate the mitochondrially encoded genetic information [2,3,4]. The integrity of mitochondrial proteins is monitored by the chaperones and AAA proteases of the mitochondrial quality control system [5]. Membrane proteins at the inner mitochondrial membrane are degraded by two membrane-bound AAA proteases [6]. Both proteases form large homo- or heterooligomeric complexes and are divided into i-AAA and m-AAA proteases [7,8,9]. The catalytic domain of the i-AAA protease faces the inter membrane space [10] and its activity is enhanced by the Mgr1 and Mgr3 proteins [11,12]. Both proteins form a subcomplex that directly binds to the i-AAA oligomer and presumably assists in recruiting substrates to the protease. The catalytic domain of the m-AAA protease faces the matrix. It consists of multiple copies of the Yta10 (Afg3) and Yta12 proteins [8]. Phb1 together with Phb2 has been shown to limit protein turnover by the m-AAA protease by offering a membrane scaffold for newly synthesized proteins [13]. Phb1 and Phb2 form a complex that binds to the m-AAA protease. How the Phb1/Phb2 complex determines proteolytic activity is currently not completely understood [14,15].

In a previous study, we found that in mitochondria of *Saccharomyces cerevisiae* (*S. cerevisiae*) gene expression is organized in large assemblies, the so-called MIOREX ^(mitochondrial organization of gene expression) complex [16]. These assemblies contain factors for mRNA maturation, mitoribosomes, chaperones, and the membrane-bound AAA proteases. Furthermore, we found several uncharacterized proteins to be part of the MIOREX complexes. The MIOREX component Mrx15 was shown to have overlapping functions with the mitoribosome receptor Mba1 [17]. Mba1 is part of the mitochondrial membrane protein insertion machinery [18,19,20,21]. Both proteins are required for membrane attachment of the mitoribosome and Cox2 membrane insertion. Therefore, co-translational translocation across the inner mitochondrial membrane is defective in the absence of Mrx15 and Mba1. 

Here we show that absence of Mrx15 renders cells resistant towards proteotoxic stress evoked by mistranslation in mitochondria. On the contrary, a yeast strain lacking Mba1 became hypersensitive against mistranslation and shows an altered translation pattern in the presence of the translational error-inducing antibiotic paromomycin. Additionally, we reveal a functional interaction between Mrx15 and the i-AAA protease regulators Mgr1 and Mgr3. We also show that Mba1 functionally cooperates with Mgr1, Mgr3, and Phb1. These results demonstrate a close connection between the mitochondrial membrane protein insertion machinery and quality control system.

## 2. Materials and Methods

### 2.1. Yeast Strains and Growth Media

All yeast strains used in this study were isogenic to either the wildtype strains W303a or BY4741. *MRX15* (*YNR040W*) was disrupted with a Kanamycin resistance cassette. The *MBA1* coding sequence was replaced with a *HIS*3 selection cassette. *MGR1* and *MGR3* were disrupted by a *URA3* or *LEU2* cassette, respectively. The *PHB1* coding sequence was exchanged by a *URA3* cassette. *MTO1* was disrupted by a *URA3* cassette.

Cells were grown at various temperatures in YP-medium (2% peptone, 1% yeast extract) in the presence of either 2% dextrose (YPD) or 2% glycerol (YPG). 

### 2.2. Paromomycin Disk Diffusion Assay

For the disk diffusion, assay cells were grown to log phase (OD_600_ 0.8–1) and plated on YPG plates. Afterwards, a sterilized filter paper was placed in the middle of the plate and 1 mM paromomycin dissolved in water was added. Plates were incubated 2–3 days at 30 °C and the size of the halo determined in comparison to the wildtype.

### 2.3. Isolation of Mitochondria

Mitochondria were isolated as described previously [22].

### 2.4. Labeling of Mitochondrial Translation Products in Organello

Isolated mitochondria were resuspended in translation buffer (0.6 M sorbitol, 150 mM KCl, 15 mM KPi, pH 7.4, 20 mM Hepes, pH 7.4, 12.67 mM MgCl_2_, 4 mM ATP, 0.5 mM GTP, 5 mM phosphoenolpyruvate, 5 mM α-ketoglutarate, 12.13 µg/mL alanine, arginine, aspartic acid, asparagine, glutamic acid, glutamine, glycine, histidine, isoleucine, leucine, lysine, phenylalanine, proline, serine, threonine, tryptophane, tyrosine, and valine, 66.67 µM cysteine, and 10 µg/mL pyruvate kinase) to 1 mg/mL. After 5 min incubation at 30 °C, 1 mM paromomycin or water was added and the mixture incubated for 30 min. Subsequently, 4 μCi [^35^S] methionine/100 µg mitochondria was added and mitochondrial translation products labelled for 20 min. A sample was taken, 8 mM cold methionine added, and the stability of labelled polypeptides checked 30 min after the labeling reaction.

## 3. Results

### 3.1. MRX15 Deletion Causes a Proteotoxic Resistant Phenotype

**Figure 1:**
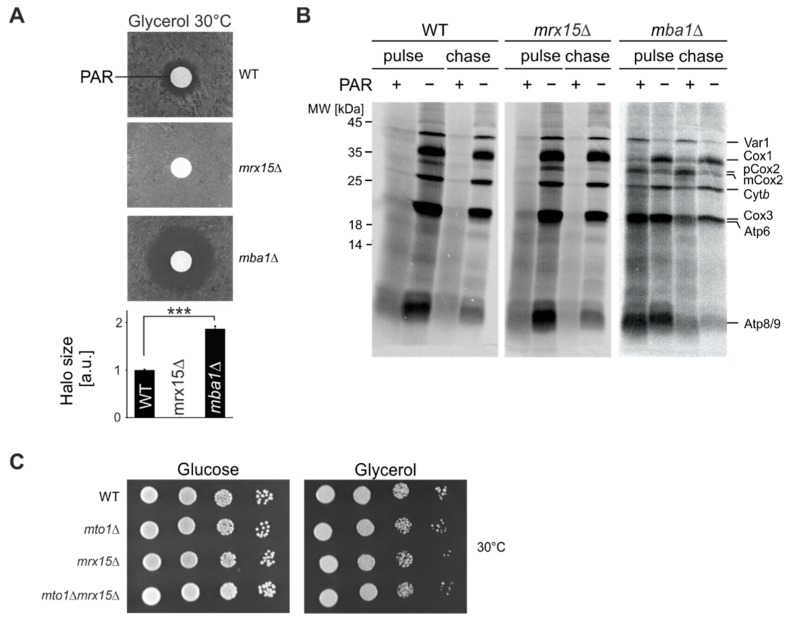
Mba1 and Mrx15 fulfil opposite roles in proteotoxic stress resistance. (**A**) Disk diffusion assay of indicated strains. Sensitivity against 1 mM paromomycin (PAR) was tested by plating cells on respiratory growth media (glycerol) and adding the antibiotic. Halo diameters of 3 independent experiments were quantified. Significance of observed changes was assessed by a student’s *t*-test (*** *p* ≤ 0.001). (**B**) In organello radiolabeling in the presence of paromomycin. Isolated mitochondria of indicated strains were incubated 30 min with 1 mM paromomycin. Afterwards, mitochondrial translation products were labelled for 20 min and the stability checked 30 min after synthesis. As control, the reaction was performed without the antibiotic. (**C**) Cells were spotted in serial 10-fold dilutions on solid media containing fermentable (glucose) or non-fermentable (glycerol) carbon sources and incubated at 30 °C. WT, wildtype.

In a previous study we showed that the MIOREX component Mrx15 (*YNR040W*) has overlapping functions with Mba1 [17] in mitoribosome membrane attachment and co-translational protein insertion. In order to find functional differences between both proteins, we evaluated how the absence of either protein affected cell growth during proteotoxic stress. Therefore, we tested sensitivity of *mrx15*∆ and *mba1*∆ mutants towards the aminoglycoside paromomycin. This antibiotic induces translational misreading by facilitating accommodation of non-cognate tRNAs, resulting in an error-prone mRNA translation in bacteria [23]. In a previous study we found that mutations of the bacterial ribosome that convey paromomycin resistance or hypersensitivity were reproduced in the *S. cerevisiae* mitoribosome [24]. These mutants showed unaffected or abolished mitochondrial protein synthesis in the presence of paromomycin, respectively. On this basis, we conclude that paromomycin directly acts on the mitoribosome, inducing translational misreading, production of non-native polypeptides and, consequently, proteotoxic stress in *S. cerevisiae* mitochondria. In a disk diffusion assay, wild type cells showed a typical halo around the filter where growth was inhibited by the presence of paromomycin (Figure 1A). Growth of the *mrx15*∆ mutant was not affected at any paromomycin concentration, while the *mba1*∆ mutant was hypersensitive towards the antibiotic. We concluded that in *mrx15*∆ mutants, proteotoxic stress tolerance is increased, and in *mba1*∆ mutants, decreased. 

Mrx15 and Mba1 directly interact with the mitoribosome and play a role in co-translational membrane protein insertion [17,19,20]. We next aimed at determining the fate of newly synthesized proteins during translation in the presence of paromomycin. We incubated isolated mitochondria from wildtype, *mrx15*∆, and *mba1*∆ strains for 30 min with paromomycin prior to radiolabeling mitochondrial translation products in organello for 20 min (Figure 1B). We followed the stability of the synthesized polypeptides for 30 min. As a control we performed the same reaction without the antibiotic. Mitochondria from the wild type and *mrx15*∆ showed impaired translation upon addition of paromomycin, because production of mature full-length proteins was strongly inhibited. As shown before, upon deletion of *MBA1*, protein synthesis was generally reduced [18,19]. The accumulation of a premature version of Cox2 (pCox2) in the absence of Mba1 has been also documented before. Upon addition of paromomycin, mitochondria lacking Mba1 retained the ability to accumulate full length polypeptides. Interestingly, in the *mba1*Δ mitochondria, the synthesis of Var1 and Cox2 was more efficient after addition of paromomycin, when compared to protein synthesis in *mba1*Δ mitochondria in the absence of paromomycin, while the production of Cox1 and Cyt*b* was reduced. Synthesis of Cox3, Atp6, Atp8, and Atp9 was unaltered in the presence of paromomycin. In summary, mitochondrial protein synthesis is inhibited in the same way in wild type and the *mrx15*Δ mitochondria by addition of paromomycin, while protein synthesis in *mba1*Δ mitochondria is mildly and differentially affected. This could be explained by either a role of Mba1 in translational accuracy or in turnover of mitochondrially encoded proteins, that in this case then fail to be degraded.

In order to test if Mrx15 is directly involved in mitochondrial translation fidelity, we tested growth in the combined absence of Mto1 and Mrx15. Mto1 modifies the wobble base of mitochondrial tRNAs and thereby alters tRNA structure, stability, and codon-anticodon interactions [25] to impact translational accuracy. However, Mto1 is only required for respiratory growth in paromomycin resistant cells [26]. Here we found that in the paromomycin resistant *mrx15*Δ mutant, *MTO1* is not required for growth on fermentative or respiratory media (Figure 1C). Accordingly, Mrx15 is most likely not directly required for translational fidelity in yeast mitochondria. In summary, we concluded that in the absence of Mrx15, cells can tolerate higher amounts of non-native polypeptides, while absence of Mba1 provokes hypersensitivity against paromomycin. Additionally, the antibiotic specifically alters the accumulation of mitochondrially encoded proteins in *mba1*Δ mitochondria.

### 3.2. MRX15 and MBA1 Functionally Interact with Regulators of the i-AAA Protease

**Figure 2:**
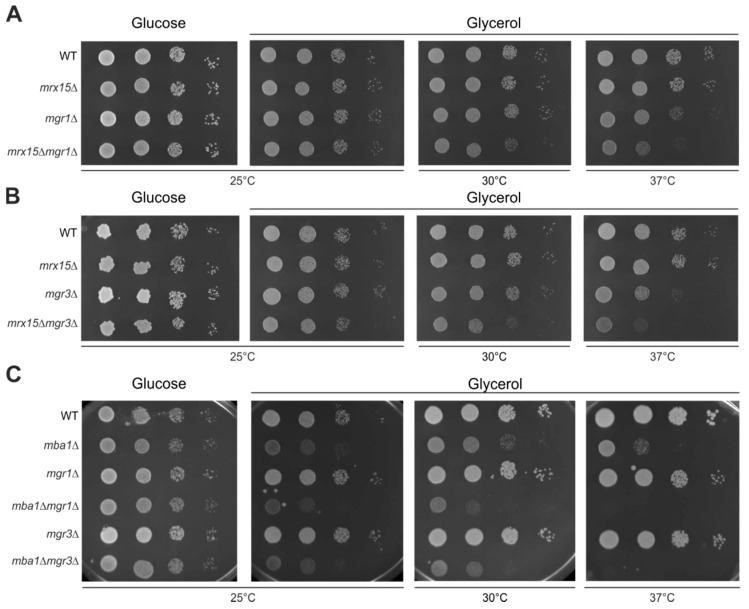
*MRX15* and *MBA1* functionally interact with *MGR1* and *MGR3*. (**A**–**C**) Serial dilution growth test on fermentable (glucose) and non-fermentable (glycerol) carbon sources of indicated strains. Cells were grown to logarithmic phase, spotted in 10-fold dilutions, and incubated at 25 °C, 30 °C and 37 °C.

Non-native mitochondrial proteins are degraded by the AAA proteases of the mitochondrial quality control system [6]. The i-AAA protease is positively regulated by the Mgr1 protein [12]. The proteotoxic stress assay suggested a function of *MRX15* in degradation of non-native mitochondrially encoded proteins. We therefore tested respiratory growth upon combined absence of Mrx15 and Mgr1. The single *mrx15*∆ and *mgr1*∆ mutants showed wild type growth upon normal, heat, and cold stress conditions on fermentative and respiratory media (Figure 2A). However, respiratory growth was slightly inhibited under normal conditions in the *mrx15*∆*mgr1*∆ mutant. This growth inhibition became more pronounced under heat stress.

Mgr3 is engaged in a complex with Mgr1 and is consequently also responsible for positively regulating the i-AAA protease [11]. We therefore assumed that *mrx15*∆*mgr3*∆ cells would behave similarly to the *mrx15*∆*mgr1*∆ mutant. Indeed, we found a growth inhibition on respiratory media upon the combined deletion of *MRX15* and *MGR3* under normal and heat stress conditions (Figure 2B). Growth on fermentative media was not affected in in any of the tested strains at all conditions.

Next, we asked whether *MBA1* also cooperates with *MGR1* and *MGR3*. Therefore, we tested growth upon the combined absence of Mba1 and Mgr1 or Mgr3, respectively (Figure 2C). As reported previously, the deletion of *MBA1* caused a slight growth defect on non-fermentable carbon sources [17,19]. This growth defect became more pronounced upon the combined deletion of *MBA1* with either *MGR1* or *MGR3*. The *mba1*Δ*mgr1*Δ and *mba1*Δ*mgr3*Δ mutants were not able to grow on non-fermentative media at increased temperatures that induce protein misfolding. 

We concluded that the regulatory proteins of the i-AAA protease Mgr1 and Mgr3, as well as Mrx15, are not strictly required for respiratory growth. However, the growth defect upon combined deletion of *MRX15* or *MBA1* with either *MGR1* or *MGR3* implies a functional interaction between these components of the mitochondrial membrane-protein insertion machinery and the quality control system.

### 3.3. Mgr1 and Mgr3 are Required during Proteotoxic Stress

**Figure 3:**
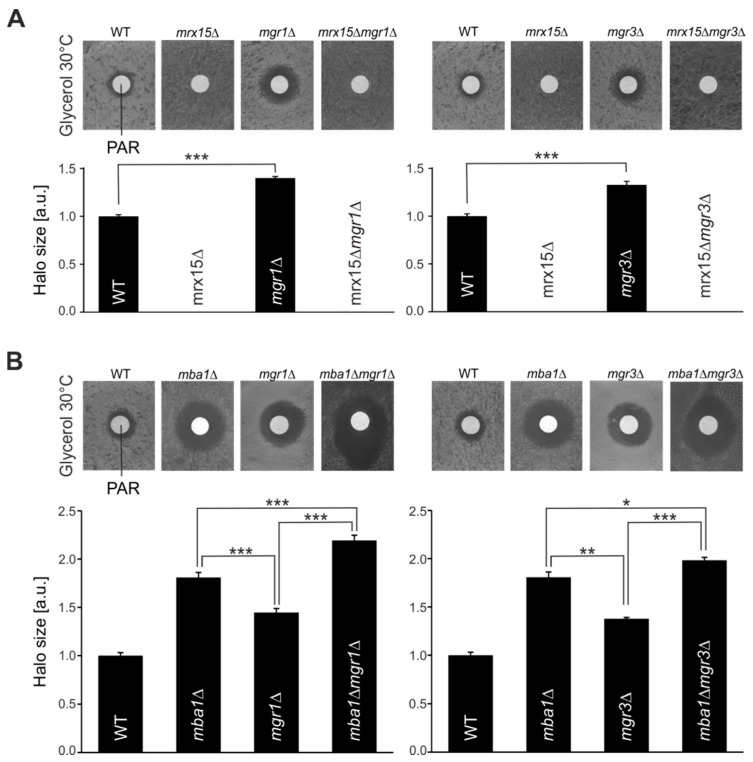
*MGR1* and *MGR3* are required during proteotoxic stress. (**A**,**B**) Disk diffusion assays of indicated strains. Sensitivity against 1 mM paromomycin (PAR) was tested by plating cells on respiratory media (glycerol) and adding the antibiotic. Halo diameters of three independent experiments were quantified. Significance of observed changes was assessed by a student’s *t*-test (* *p* ≤ 0.05, ** *p* ≤ 0.01, *** *p* ≤ 0.001).

Mrx15 and Mba1 have overlapping functions. Nevertheless, in the absence of either protein, yeast cells showed the opposite growth behavior under proteotoxic stress conditions (Figure 1A). We therefore determined how the absence of Mgr1 and Mgr3 would affect growth in the presence of paromomycin. As shown before, upon deletion of *MRX15*, cells became resistant against paromomycin, while the *mgr1*∆ and *mgr3*∆ cells became hypersensitive against the antibiotic (Figure 3A). Surprisingly, both double mutants, *mrx15*∆*mgr1*∆ and *mrx15*∆*mgr3*∆, were resistant to paromomycin, behaving identically as the *MRX15* single deletion strain. 

Next, we investigated how the combined absence of Mgr1 or Mgr3 together with Mba1 affected growth during proteotoxic stress (Figure 3B). As shown previously (Figure 1A and Figure 3A), the absence of Mgr1, Mgr3, and Mba1 caused hypersensitivity against paromomycin. However, we found that sensitivity of *mba1*∆*mgr1*∆ and *mba1*∆*mgr3*∆ mutants against proteotoxic stress was further increased when compared to the respective single deletion mutants. These results show an additive effect on the proteotoxic stress sensitivity upon combined absence of Mba1 with either Mgr1 or Mgr3, suggesting a role of Mba1 in the degradation of non-native polypeptides.

In summary, we show here that in the absence of Mgr1 and Mgr3, yeast cells become hypersensitive against proteotoxic stress, which could be explained by defects to deliver substrates to the i-AAA protease. This sensitivity is increased upon combined absence with Mba1. However, the necessity of Mgr1 and Mgr3 during proteotoxic stress is circumvented in the absence of Mrx15, suggesting that Mrx15 plays a pivotal role in handling non-native proteins. 

### 3.4. MRX15 is Specifically Linked to the i-AAA Protease

**Figure 4:**
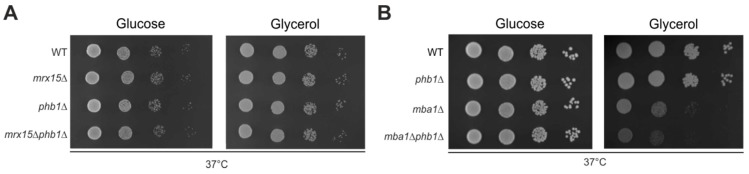
*MBA1* functionally interacts with *PHB1*. (**A**) and (**B**) Serial dilution growth test on fermentable (glucose) and non-fermentable (glycerol) carbon sources of indicated strains. Cells were grown to logarithmic phase, spotted in 10-fold dilutions, and incubated at 37 °C.

Because we found a functional connection of *MRX15* with the regulators of the i-AAA protease (Figure 2A,B), we asked whether *MRX15* also functionally interacts with *PHB1*, a regulator of the m-AAA protease [13]. The *phb1*∆ mutant showed normal growth under heat stress conditions (Figure 4A,B). Contrary to the regulators of the i-AAA protease, the combined deletion of *MRX15* and *PHB1* did not cause a growth defect on respiratory media (Figure 4A). Thus, *PHB1* was not required for growth upon heat stress conditions or membrane-protein insertion defects. Since both *MRX15* and *MBA1* showed a functional interaction with the i-AAA protease regulators *MGR1* and *MGR3*, we tested if *MBA1*, contrary to *MRX15*, functionally interacts with *PHB1*. We found that in the *mba1*Δ*phb1*Δ mutant, respiratory growth was decreased when compared to the respective single deletion mutants (Figure 4B). 

We concluded that Mrx15 is functionally interacting with the Mgr1/Mgr3 complex but not with the Phb1/Phb2 complex. On the other hand, Mba1 cooperates with the regulators of the m-AAA and i-AAA protease. 

## 4. Discussion

In this study, we tested the effects of proteotoxic stress in the absence of components of the mitochondrial membrane protein insertion machinery and regulators of the mitochondrial quality control AAA proteases. Strikingly, the absence of the ribosome receptor Mrx15 provoked a dominant resistance against paromomycin. Additionally, *MRX15* functionally interacts with the regulators of the i-AAA protease *MGR1* and *MGR3*. These results suggest a role for Mrx15 in the degradation of non-native mitochondrial polypeptides. Likewise, *MBA1* displays a genetic interaction with genes implicated in mitochondrial protein turnover. This together suggests that membrane insertion of mitochondrially encoded proteins and their turnover is functionally coupled and that this coupling is important for mitochondrial proteostasis.

Yeast cells lacking Mrx15 showed a dominant resistance against paromomycin. This could be explained by a direct role of Mrx15 in degradation of non-native polypeptides caused by the antibiotic or a general drug resistance in the absence Mrx15. At this moment it is not possible to discriminate between these possibilities. The functional cooperation with *MGR1* and *MGR*3 links *MRX15* to the mitochondrial quality control system and is therefore in line with the first alterative. However, it was shown that disruption of the *OXA1* insertase causes a multiple drug resistance [27]. This study uncovered a regulatory pathway that couples mitochondrial function to expression of multi-drug-resistance genes. Future studies will have to address if the paromomycin resistance in *mrx15*∆ mutants is linked directly to protein degradation or the same multi-drug-resistance pathway that is activated in *oxa1*∆ cells. 

Specific substrates at the inner mitochondrial membrane, whose degradation depends on the Mgr1/Mgr3 complex, are currently not known. The functions of Mgr1 and Mgr3 have been studied on imported model substrates [11,12]. Moreover, a recent study showed that Mgr1 and Mgr3 are responsible for the degradation of outer membrane proteins [28]. Here we provide evidence that the Mgr1/Mgr3 complex is involved in turnover of mitochondrially encoded proteins. At the moment it is not clear whether Mrx15 fulfills a general role in mitochondrial membrane protein insertion or is specifically required for Cox2 biogenesis [17]. In the latter case, the functional interaction of *MRX15* with *MGR1* and *MGR3* would suggest Cox2 as candidate substrate for the Mgr1/Mgr3 complex and degradation by the i-AAA protease. This is in line with several studies suggesting Cox2 as a i-AAA protease substrate [29,30,31]. 

The functional interaction of *MRX15* was specific for the regulators of the i-AAA protease. As discussed above, this could be related to the different substrates of the m-AAA and i-AAA proteases. Furthermore, the Phb1 and Phb2 regulators are universally conserved, whereas Mgr1, Mgr3 and Mrx15 are confined to fungi [6]. This observation could hint at a common function of the three proteins, as revealed by their genetic interactions showing redundancy between the factors. Phb1 alone was not required under any of the tested stress conditions in this study. Only the combined absence of Phb1 and Mba1 caused a growth inhibition on respiratory growth media. Consequently, Phb1 is required upon insertion defects of mitochondrially encoded proteins [18,19,20,21]. The Phb1/Phb2 complex has been suggested to serve as a membrane-bound chaperone protecting polypeptides from degradation by the m-AAA protease [13]. However, the specific function of the Phb1/Phb2 complex is still under debate and other functions have been proposed [14]. 

Mba1 was initially discovered as multi-copy bypass of the m-AAA protease [32], suggesting a role of Mba1 in degradation of mitochondrial proteins. The connection between Mba1 and the regulators of the membrane-bound AAA proteases supports a role of this ribosome receptor in regulation of protein degradation. Such a function is supported by data reported here that the absence of Mba1 sensitizes cells towards proteotoxic stress. Moreover, absence of Mba1 allowed the accumulation of full-length mitochondrial polypeptides in the presence of paromomycin (Figure 1B). In the *mrx15*Δ mutant and wild type mitochondria, full-length mitochondrial proteins were not detected in the presence of paromomycin. This result suggests an active function of Mba1 in surveillance of mitochondrial proteins. Such a surveillance would be circumvented in the absence of Mba1, allowing the accumulation of non-native, but full-length polypeptides. 

Surprisingly, the ribosome receptors Mrx15 and Mba1 seem to carry out different roles in protein surveillance, as their absence causes different sensitivity against proteotoxic stress and they cooperate with different subsets of the membrane-bound AAA proteases. How are the functions of Mrx15 and Mba1 in co-translational membrane protein insertion related to the here-proposed role in degradation of mitochondrial proteins? It is tempting to speculate that surveillance of mitochondrially encoded proteins by the mitochondrial quality control system occurs during or shortly after protein synthesis. At this point, Mba1 and Mrx15 could play an active role in this process. In the MIOREX complex, the m-AAA and i-AAA proteases are found together with the regulators of the AAA proteases and Mrx15 [16,17]. Accordingly, the components of the membrane-bound mitochondrial quality control system are in proximity to the mitochondrial ribosome and Mrx15, as well as Mba1. The bacterial homolog of the m-AAA protease, FtsH, directly interacts with YidC and nascent polypeptide chains [33]. YidC is the bacterial homolog of the mitochondrial membrane protein insertase Oxa1 [34]. Therefore, bacterial quality control by membrane-bound AAA proteases occurs at a similar step as suggested by the genetic interactions of the ribosome receptors and quality control components reported in this study. Future studies are required in order to determine how an early quality control mechanism cooperates with co-translational membrane protein insertion on a molecular level.
